# Assessment of the Breast Density Prevalence in Swiss Women with a Deep Convolutional Neural Network: A Cross-Sectional Study

**DOI:** 10.3390/diagnostics14192212

**Published:** 2024-10-03

**Authors:** Adergicia V. Kaiser, Daniela Zanolin-Purin, Natalie Chuck, Jennifer Enaux, Daniela Wruk

**Affiliations:** 1 Faculty of Medical Sciences, Private University in the Principality of Liechtenstein (UFL), 9495 Triesen, Liechtenstein; daniela.purin@ufl.li (D.Z.-P.); jennifer.enaux@srrws.ch (J.E.); 2 St. Gallen Radiology Network, Cantonal Hospital of St. Gallen, 9007 St. Gallen, Switzerlanddaniela.wruk@srrws.ch (D.W.); 3 St. Gallen Radiology Network, Grabs Hospital, 9472 Grabs, Switzerland

**Keywords:** breast density assessment, breast density distribution, deep convolutional neural network, prevalence of dense breasts, Swiss population

## Abstract

**Background/Objectives:** High breast density is a risk factor for breast cancer and can reduce the sensitivity of mammography. Given the influence of breast density on patient risk stratification and screening accuracy, it is crucial to monitor the prevalence of extremely dense breasts within local populations. Moreover, there is a lack of comprehensive understanding regarding breast density prevalence in Switzerland. Therefore, this study aimed to determine the prevalence of breast density in a selected Swiss population. **Methods:** To overcome the potential variability in breast density classifications by human readers, this study utilized commercially available deep convolutional neural network breast classification software. A retrospective analysis of mammographic images of women aged 40 years and older was performed. **Results:** A total of 4698 mammograms from women (58 ± 11 years) were included in this study. The highest prevalence of breast density was in category C (heterogeneously dense), which was observed in 41.5% of the cases. This was followed by category B (scattered areas of fibroglandular tissue), which accounted for 22.5%. **Conclusions:** Notably, extremely dense breasts (category D) were significantly more common in younger women, with a prevalence of 34%. However, this rate dropped sharply to less than 10% in women over 55 years of age.

## 1. Introduction

Breast density refers to the amounts of fibroglandular tissue and fat in the breast [1]. Fibroglandular tissue appears radiodense in mammography, while fat tissue appears radiolucent [2]. Since breast tumors also appear radiodense in mammography, high breast density may hinder tumor detection because of a masking effect, thus limiting the sensitivity and specificity of mammography [3,4]. Moreover, studies indicate breast density as an independent risk factor for the development of breast cancer, reporting up to a six-fold increased breast cancer risk in dense breasts compared to fatty breasts [2,5,6,7]. In addition, high breast density is also associated with increased false negative and interval cancer rates [2,5,8,9,10].

In clinical settings, the American College of Radiology Breast Imaging Reporting and Data System (ACR BI-RADS) density score, which is based on the radiologist’s visual interpretation, is the most commonly used breast density assessment. However, this visual assessment can lead to poor intra- and inter-reader agreement [11]. Especially for clinically relevant actions, the misclassification of breast density could lead to the overestimation or underestimation of women’s predictive risks [12,13,14]. To overcome these limitations, the use of automated volumetric breast density assessments has been introduced [15]. However, while volumetric assessments are correlated with cancer risk, they have been reported to underestimate the masking effect and show disagreements when compared with ACR BI-RADS 5th Edition measurements by radiologists [16,17]. Consequently, the establishment of a standardized classification system for mammographic breast density is essential for clinical practice, as it allows for consistent, reproducible recommendations for supplemental investigation and ensures adequate management for women at high risk of developing breast cancer [12,13,14].

Recently, advances in artificial intelligence (AI) have enabled the development of algorithms that can be used to interpret complex patterns in radiological images [15,18]. In mammography, studies have shown that AI software can achieve classification accuracies for breast density comparable to those of experts in the field [19,20,21]. In the United States, Mohamed et al. (https://www.ncbi.nlm.nih.gov/pmc/articles/PMC5774233/ accessed on 1 June 2024) and Lehman et al. (ResNet-18 with PyTorch v 0.31) developed software using a deep convolutional neural network (DCNN) that showed good agreement in breast density assessment compared with human readers [19,21]. Similarly, Ciritsis et al. reported an average accuracy of 91% for breast density classification using DCNN software (b-box v 1.1.0) trained and validated according to the ACR BI-RADS Atlas [20].

High breast density has been reported in over 40% of the screening population aged 40 to 74 years old in the United States [22]. Youn et al., using a fully automated volumetric assessment, reported a dense breast rate of 83.2% among women participating in the Korean national screening program [23]. Since the prevalence of breast density may be influenced by many factors, including age, body mass index (BMI), ethnicity, menopausal status, and hormone replacement therapy [24,25,26,27,28], the results may not be generalizable to different populations. Furthermore, the limited understanding of breast density prevalence in regional screening populations underscores the urgent need for further research. Therefore, this study aimed to determine the prevalence of breast density in a selected Swiss population. To mitigate the potential variability in breast density classifications by human readers, this study utilized commercially available deep convolutional neural network breast classification software.

## 2. Materials and Methods

### 2.1. Study Design

This study was approved by the Ethics Committee of Eastern Switzerland (EKOS 21/154), which waived the requirement for informed consent.

We conducted a retrospective analysis of a dataset of consecutive mammographic images from women 40 years and older who underwent diagnostic mammography exams and opportunistic mammography screening at the Radiology Center of Cantonal Hospital in St. Gallen, Switzerland. In this study, the term “opportunistic mammography screening” refers to mammography performed on asymptomatic women to identify breast cancer outside the frame of a national screening program. The national breast cancer screening program was implemented in the cantons of St. Gallen and Graubünden in 2010 and has been running alongside the opportunistic mammography program. Since the national breast cancer program was launched, the participation rate has gradually increased, reaching nearly 50% by 2018 [29]. Data collection was performed by the first author (AK) from February 2022 to January 2023. This study is reported in accordance with the Strengthening the Reporting of Observational Studies in Epidemiology (STROBE) Guidelines [30].

### 2.2. Study Population

This study analyzed 10,574 mammography exams (Full Film Digital Mammography and Film Mammography) performed between 1 January 2009 and 31 May 2014 at the reported breast cancer screening facility. The electronic radiology report was searched for demographic data according to the inclusion and exclusion criteria. We included film mammography because it was still being used during the study period. Mammography exams from women with a personal history of breast cancer, breast augmentation, breast surgery, breast radiotherapy, and chest radiation and those from women under the age of 40 years old were excluded from this study. These exclusion criteria were applied to avoid the influence of mammography findings on breast density assessment, as well as to obtain a sample of women with average breast cancer risks. In addition, we excluded women younger than 40 years old because this study aimed to focus on asymptomatic women.

The sample size for this study was determined based on the following factors: an expected minimum effect of age on the prevalence of breast density of about 5–10% [1], a minimum reported prevalence of 10% of breast densities A and D in the literature [1,22], a power of 80%, and a confidence level of 95%. The power calculations recommended a minimum sample size of around 1500 participants. Because of the strict inclusion and exclusion criteria of the study and to ensure a balanced representation of all four breast density categories in the study population, the sample size was increased to approximately 5000 participants.

### 2.3. Breast Density Assessment

This study utilized commercially available breast density classification software (b-box v 1.1.0, b-rayZ AG, Schlieren, Switzerland), including a machine learning algorithm based on a deep convolutional neural network (DCNN) trained and validated in accordance with the ACR BI-RADS 5th Edition. The software involved a DCNN with 11 convolutional layers and 3 fully connected layers. It was trained and validated using 20,578 mammography images from 5221 women. Detailed information about the training, validation, and architecture of the software can be found in the comprehensive description provided by Ciritsis et al. [20].

For each subject, the standard craniocaudal and mediolateral oblique views of both breasts acquired with the Siemens Mammomat Inspiration and PRIME^®^ (Healthineers AG, Erlangen, Germany) units were manually retrieved from the local picture archiving and communication system (PACS), sorted according to the study criteria listed above, and imported into the software. The software analyzed the imported mammography image and assigned the breast density to a category in the ACR BI-RADS 5th Edition. The ACR BI-RADS classifies breasts as almost entirely fatty (category A), scattered areas of fibroglandular tissue (category B), heterogeneously dense (category C), and extremely dense (category D) [1]. Breast density categorized as C and D are defined as dense, whereas A and B are defined as nondense. The software produced .csv files containing raw data, including patient names, patient identification numbers, dates of birth, four standard mammography projections, and breast density category scores. The examination date was determined by assuming the first day of each month as the examination date. All data were organized in spreadsheets, and any repeated views were removed.

### 2.4. Statistical Analyses

Descriptive statistics were used to summarize and describe categorical data. In particular, counts and percentages were reported for each selected age range of the population. The variables analyzed were breast density distribution in the following four categories: A, B, C, and D according to the ACR BI-RADS 5th edition. The breast density distribution was represented by the dichotomous variables dense and nondense. Age was distributed in the following nine groups: group 1: 40–44; group 2: 45–49; group 3: 50–54; group 4: 55–59; group 5: 60–64; group 6: 65–69; group 7: 70–74; group 8: 75–79; and group 9: 80 plus. *p*-Values < 0.05 were considered statistically significant and reported as two-sided confidence intervals. The SPSS software package (SPSS version 28, IBM Corp., Armonk, NY, USA) was used to perform all statistical analyses.

## 3. Results

### 3.1. Study Population

**Figure 1:**
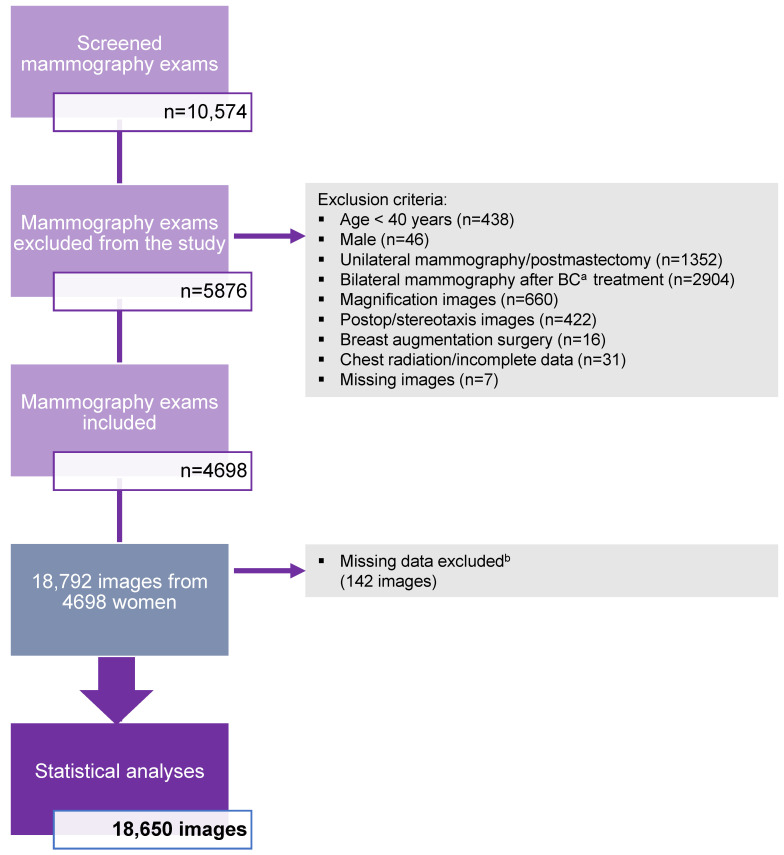
Study population flowchart illustrating the specific criteria for exclusion in the study. A total of 4698 mammography exams were included in the study. ^a^ BC = breast cancer. ^b^ Missing data excluded = breast density score not generated by the software.

This study analyzed a total of 10,574 mammography exams from women aged 40 years and older who underwent a mammography exam at a breast screening facility from 2009 to 2014. A total of 5876 mammography exams did not meet the inclusion criteria and were excluded from the study. Approximately 70% of the mammography exams excluded from the study were from confirmed breast cancer cases (*n* = 4256). A total of 4698 mammography exams (*n* = 18,792 images) of women aged 40 years and older were enrolled in the study. The software was unable to generate breast density scores for two mammography exams (*n* = 8) and a few of the single views (*n* = 134), which represented 0.8% of the total dataset. These failures (*n* = 142) were excluded from the statistical analyses as missing data. Thus, software calculations of breast density were based on four images for 99.2% of the data from all examinations. The final statistical analyses were performed on 18,650 images. These results are presented in a flowchart (Figure 1).

The mean age of the participants was 58 years old with a standard deviation of 11 years (age range: 40 to 95 years). The distribution of the age groups shows that the majority of mammography exams were performed on women between the ages of 40 and 74 years, representing approximately 92% of the total examinations analyzed in this study. Women aged 50 to 74 years old, which is the age range for eligibility for screening in the eastern part of Switzerland, accounted for 69% of the total study population. The demographic characteristics of the study population are summarized in Table 1.

### 3.2. Breast Density Distribution

**Figure 2:**
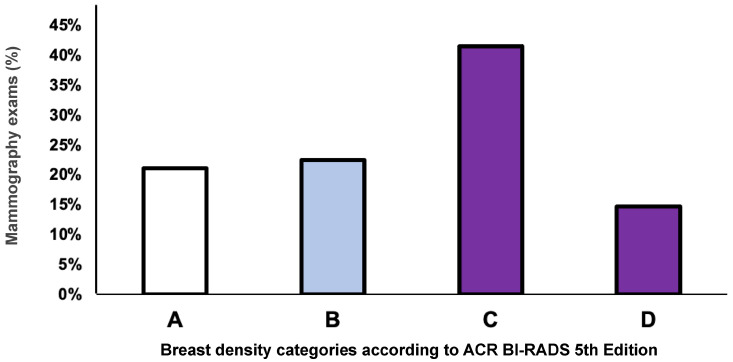
The overall distribution of the breast density categories according to the American College of Radiology Breast Imaging Reporting and Data System (ACR BI-RADS) 5th Edition. A = almost entirely fatty; B = scattered areas of fibroglandular tissue; C = heterogeneously dense; D = extremely dense. The X-axis represents breast density categories, and the Y-axis represents the frequency of mammography as a percentage.

Among all mammography exams, the highest prevalence was observed for category C—heterogeneously dense (41.5%) followed by category B—scattered areas of fibroglandular tissue (22.5%), category A—almost entirely fatty (21.2%), and category D—extremely dense (14.7%). For the overall cohort, 56% of all mammograms analyzed were categorized as dense breasts, which includes categories C (heterogeneously dense) and D (extremely dense) combined, as shown in Figure 2.

### 3.3. Breast Density Categories According to the ACR BI-RADS 5th Edition by Age Group

**Figure 3:**
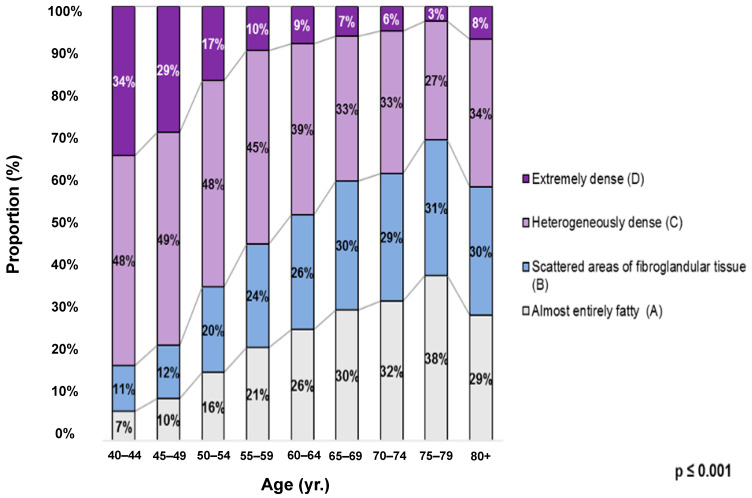
Distribution of breast density by age group among the study population according to the American College of Radiology Breast Imaging Reporting and Data System (ACR BI-RADS) 5th Edition. A = almost entirely fatty; B = scattered areas of fibroglandular tissue; C = heterogeneously dense; D = extremely dense. (yr.); ages grouped in years. *p*-Value (*p* < 0.001) refers to the distribution of breast density categories across different age groups. The younger age groups show significant variations in breast density distribution when compared to each other, with categories C (heterogeneously dense) and D (extremely dense) being predominant (*p* < 0.001).

When the four breast density categories were stratified by age group, the prevalence remained significantly elevated in the heterogeneously dense category (category C). A subgroup analysis comparing age groups revealed significant differences in the breast density distribution. The younger age groups (40–44, 45–49, and 50–54) showed significant differences in breast density distributions compared to each other, indicating more significant variability and a greater predominance of dense categories in the younger groups (*p* < 0.001), with categories C and D being predominant. However, for the age group 65–69 years and older, there were no significant differences in this age group (*p* > 0.005), indicating a more even distribution of breast density categories, with categories A and B being more prevalent. These results are shown in Figure 3.

### 3.4. Distribution of the Dichotomous Categories Nondense and Dense by Age Group

**Figure 4:**
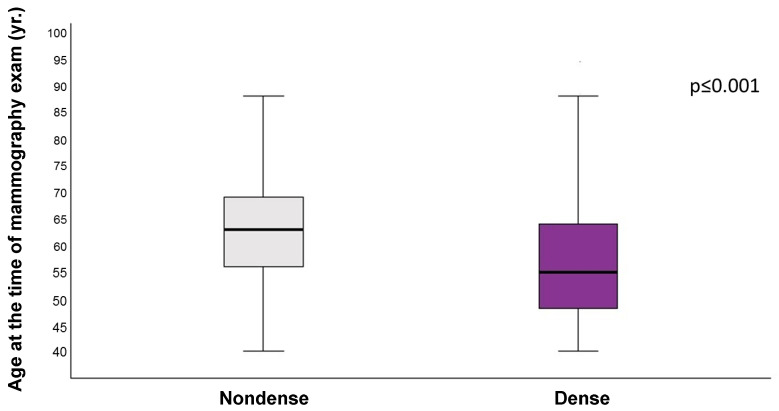
Distribution of the dichotomous categories of nondense and dense by age group, according to American College of Radiology Breast Imaging Reporting and Data System (ACR BI-RADS) 5th Edition. Nondense: breast density categories A and B combined. Dense: breast density categories C and D combined. *p*-Value (*p* < 0.001) refers to the distribution of dense and nondense categories across age groups. The X-axis represents dense and nondense categories. The Y-axis represents the age of women at the time of the mammography exam. This Figure shows that women assigned to dense categories were younger than those assigned to nondense categories.

The results of the quantitative analysis of the age distributions in relation to the dichotomous categories of breast density (categories dense and nondense) are presented. The data indicate that women with dense breasts (categories C and D, combined) are, on average, younger (57 years ± 11 years) than women with nondense breasts (categories A and B, combined), whose average age was 63 years ± 9 years (*p* < 0.001). These results demonstrate that age and breast density are closely related, with younger women tending to have denser breast tissue. The results are presented in Figure 4.

## 4. Discussion

In our cohort study, we found that among local women who met the study criteria, the highest prevalence of breast density was observed for category C (heterogeneously dense) at 41.5%, followed by category B (scattered areas of fibroglandular tissue) at 22.5%, category A (almost entirely fatty) at 21.2%, and category D (extremely dense) at 14.7%. An in-depth analysis of the breast density distribution across age groups revealed that the prevalence of the extremely dense category (category D) decreased from a maximum of 34% in women aged 40–44 to 10% or less in women older than 55. For women of screening age, which is from ages 50 to 74 years old in the eastern part of Switzerland, the prevalence of dense breasts (categories C and D, combined) decreased from 65% to 39% in the study population, and the prevalence of extremely dense breasts (category D) decreased from 17% to 6% in the same age group. Since nearly 70% of the mammography exams included in this study were from women eligible for breast cancer screening, the high proportion of dense breasts among women in the younger age groups but eligible for screening may affect mammography performance, which emphasizes the need to develop age-appropriate and density-adjusted screening strategies.

It is challenging to compare the prevalence of breast density found in our study with the prevalence reported in the literature because of the diversity of breast density assessment methods and different populations. Our study estimated the prevalence of breast density comparable to the prevalence reported by few studies conducted in local populations in Asia [23,31]. Jo at al., using the BI-RADS 4th Edition, reported that more than half of Korean women aged 40 years and older presented dense breasts [31]. Youn et al., by analyzing breast density in Korean women by fully automated volumetric assessment, showed an 83.2% rate of dense breasts among women who participated in the national screening program [23]. Considering both breast density and age, the distributions of the dense and nondense categories reported in our study confirm a progressive decrease in breast density with age, which is consistent with the current literature [22,28,31].

In comparison to studies in Western women, this study found a higher rate of dense breasts. For instance, Sprague et al. report a 43% prevalence of dense breasts in women aged 40 to 74 years in the United States [22], while the current study found an even higher rate. This difference may be due to the fact that the AI software used in this study was trained to assess breast density according to the ACR BI-RADS 5th Edition. Previous studies have shown that the two different mammographic density measurements used in the 4th and 5th Editions of the BI-RADS Atlas do not produce identical results and that breast density assessment with the 5th Edition yields higher rates of dense categories compared to the 4th Edition [14,32]. Since the BI-RADS 5th Edition has revised the mammographic breast density categories by emphasizing the masking effect of dense fibroglandular tissue and its potential to hide underlying cancer [1], more mammography exams might be classified as dense breasts if there is localized dense tissue that would have been considered fatty breasts according to the percentage quartile assessment.

When comparing our results with those of Lehman et al., who analyzed breast density prevalence in women using similar AI software trained with the ACR BI-RADS 5th Edition, the prevalence reported in our study was higher [21]. This could be explained by the fact that women in our study were recruited outside a population-based screening. Thus, there may be an overrepresentation of symptomatic or high-risk women, which could be associated with a higher prevalence of dense breasts. In addition, the standardization of the readings may have affected the distribution of the density within the population [16]. Studies report increased breast density prevalence with the use of two automated volumetric assessment methods compared to a visual radiologist’s assessment [33,34].

Nevertheless, some limitations should be acknowledged while interpreting our results. This study was conducted at one institution with all mammography performed by a single mammography vendor at a single radiology facility, which could lead to a loss in the generalizability of the results to mammography acquired from other vendors. Further, in the retrospective setting of the study, we did not have access to other factors that are associated with breast density, such as BMI, ethnicity, menopausal status, and the use of menopausal hormone therapy. When the mammography exams were collected during the study period, detailed information on breast cancer risk factors, other than age and personal history of breast cancer, was either unavailable or insufficiently detailed in the radiologist’s reports. Although, these factors may potentially affect breast density prevalence, the strongest associations have been observed for age and BMI [28]. Hence, caution should be taken when extrapolating the results of this study to populations that vary markedly in such characteristics. Moreover, factors such as breast surface area, breast volume, compression force, and breast tissue blood perfusion, which may influence the assessment of breast density [35,36], were also not included in the analysis in this study. Additionally, prior research has shown that breast compression can affect the accuracy of breast density measurements obtained through automated volumetric methods [37]. In mammography, an adequate level of breast compression is crucial for ensuring high-quality imaging, reducing tissue overlap and minimizing discomfort for women undergoing breast examinations. Most studies exploring the relationship between breast compression and breast density assessment have focused on automated volumetric techniques [37,38,39,40]. However, variations in compression force protocols across different screening programs may contribute to inconsistencies in breast density evaluations. The degree of compression applied can vary among individuals and even among repeated screenings of the same woman. This variability can affect the appearance of breast tissue, potentially leading to either underestimation or overestimation of breast density. Consequently, these inconsistencies may distort density assessment outcomes, affecting screening accuracy and personalized screening recommendations. Therefore, the implementation of standardized compression force protocols in mammography is essential for enhancing diagnostic precision and consistency across screening practices [37,38,39,40]. Since our study was conducted within the same institution, by following internal guidelines for compression, we expect that this factor had minimal influence on the assessment of the prevalence of density categories in the study population. Another limitation of our study is that the software utilized does not provide a 100% accurate classification of breast density and a residual variability of up to 9% in the binary classification cannot be excluded [20]. While AI tools have shown promise in improving the accuracy and efficiency of density assessments, they may exhibit some variability in their performance depending on the quality and resolution of the imaging, training dataset used, and inherent limitations of the algorithm’s design. This can lead to discrepancies in breast density assessments across different screening programs. Therefore, it is important to acknowledge the potential limitations of AI tools and the need for continuous refinement of AI algorithms to ensure more reliable and accurate breast density assessments [15,18].

Despite the absence of data on potential confounders, our study provides a standardized assessment of breast density and confirms a strong association between breast density and age, aligning with established research [22,28,31]. Additionally, the use of breast density classification software allows for analysis of a large dataset of mammography exams, significantly reducing the human workload and improving reproducibility. Therefore, our findings may have implications for the planning of breast cancer screening programs, in which the prevalence of breast density in women eligible for the screening must be addressed. The high prevalence of dense breasts in younger age groups reported in this study highlights the necessity of developing age-appropriate and density-adjusted screening strategies. Accordingly, tailoring screening approaches based on age and breast density, alongside educating women about their breast density, could enhance early detection and screening efficacy. Further, women aged 50 to 74 years old with the breast density categories heterogeneously dense and extremely dense breasts and, thus, eligible for the mammographic screening program, might require additional resources to cover supplemental screening. Currently, no supplemental imaging modality is considered standard of care for women with dense breasts in Switzerland and, further, clinical society guidelines are unclear about the optimal supplemental modality. The European Society of Breast Imaging (EUSOBI) recommended the use of magnetic resonance imaging (MRI) in women with extremely dense breasts (D category) [41], while the US Preventive Services Task Force (US Task Force) still found insufficient evidence to recommend any particular supplemental modality for dense breasts. Nevertheless, the US Task Force acknowledges the limitations of mammography in dense breasts and recommends that women be informed during the decision-making process [42,43]. Moreover, a recent systematic literature review identified that MRI has an improved cancer detection rate in women with extremely dense breasts (D category) compared to handheld ultrasound, automated whole-breast ultrasound, and digital breast tomosynthesis (DBT) [44]. However, because of the lack of implementation of supplemental MRI, more studies are needed to consolidate its recommendation [45].

As increasing attention is directed toward personalized screening, the need to develop an optimal risk assessment model for breast cancer that can be integrated into population-level screening programs becomes crucial. However, current risk prediction models require substantial data, posing significant challenges to their integration into routine screening practices [46]. Several studies have emphasized the potential role of breast density in predicting breast cancer risk [3,8,9,47]. Other mammographic features, such as parenchymal texture, have improved the predictive value of breast cancer risk assessments and have shown a close relationship with breast density [48,49]. Further, integrating DCNN into the parenchymal texture-based model has shown potential for enhancing breast cancer risk stratification [50]. Consequently, the use of parenchymal texture evaluations and breast density scores from available mammography in breast cancer screening has the potential to enhance breast cancer risk stratification and could be seamlessly integrated into population-based screening [51,52].

Finally, future studies will assess the prevalence of breast density in other regions of the country to provide a comprehensive picture. Through rigorous studies with larger datasets and a commitment to addressing challenges and limitations, AI can be incorporated into clinical practice to standardize breast density assessments, personalize treatment, and, ultimately, improve patient outcomes in breast screening.

## 5. Conclusions

This study assessed breast density in a selected population of Switzerland using a deep convolutional neural network to standardize the classification according to the ACR BI-RADS 5th Edition. We estimated a prevalence for dense breasts of 56% in women aged 40 years and older in the local population and a dependence of the distribution on age. The prevalence of extremely dense breasts was significantly higher in younger women (34%) and decreased to less than 10% in women over 55 years of age. The high prevalence of dense breast tissue in young women undergoing screening may impact the accuracy of mammography, highlighting the importance of developing screening approaches tailored to age and breast density.

## Figures and Tables

**Table 1:** Demographic characteristics of the study population.

Participants Age at the Time of Mammography Exam	Mammography Exams ^b^ (n = 4698 Women)
Average age (yr.) ^a^	58 ± 11 ^c^
Age range (yr.) ^a^	40–95
Age group (yr.) ^a^	Frequency (%) ^d^
40–44	9.2
45–49	14.5
50–54	15.5
55–59	15.2
60–64	16.2
65–69	13.0
70–74	8.7
75–79	5.0
≥80	2.7

^a^ (yr.) = Age in years. ^b^ Mammography exams per women. ^c^ Standard deviation of the mean age. ^d^ (%) = Frequency of participants within each age group among a total of 4698 examinations.

## Data Availability

The datasets produced and analyzed in this study are not publicly accessible due to IRB and institutional restrictions but may be available upon request from the corresponding author.
